# Fibromatosis-like carcinoma-an unusual phenotype of a metaplastic breast tumor associated with a micropapilloma

**DOI:** 10.1186/1477-7819-5-24

**Published:** 2007-02-27

**Authors:** Bharat Rekhi, Tanuja M Shet, Rajan A Badwe, Roshni F Chinoy

**Affiliations:** 1 Department of Pathology, Tata Memorial Hospital, Parel, Mumbai, India; 2 Department of Surgical Oncology, Tata Memorial Hospital, Parel, Mumbai, India

## Abstract

**Background:**

Fibromatosis-like metaplastic carcinoma is a newly described metaplastic breast tumor, literature on which is still evolving.

**Case presentation:**

A 77-year-old lady presented with a 2 × 2 cm mass with irregular margins in the upper and outer quadrant of left breast. Fine needle aspiration cytology (FNAC) from the lump was inconclusive. A lumpectomy was performed and sent for frozen section, which revealed presence of spindle cells showing mild atypia in a sclerotic stroma. The tumor cells revealed prominent infiltration into the adjacent fat. A differential diagnosis of a low-grade sarcoma vs. a metaplastic carcinoma, favoring the former, was offered. Final histology sections revealed an infiltrating tumor with predominant spindle cells in a collagenous background, simulating a fibromatosis. Adjacent to the tumor were foci of benign ductal hyperplasia and a micropapilloma. Immunohistochemistry (IHC) showed diffuse co-expression of epithelial markers i.e. cytokeratins (CK, HMWCK, CK7) and EMA along with a mesenchymal marker i.e. vimentin in the tumor cells. Myoepithelial markers (SMA and p63) showed focal positivity. A diagnosis of a low-grade fibromatosis-like carcinoma breast associated with a micropapilloma was formed.

**Conclusion:**

Fibromatosis-like carcinoma is a rare form of a metaplastic breast tumor. This diagnosis requires an index of suspicion while dealing with spindle cell breast tumors. The importance of making this diagnosis to facilitate an intra operative surgical planning is marred by diagnostic difficulties. In such cases, IHC is imperative in forming an objective diagnosis.

## Background

Metaplastic breast tumors exhibit a wide morphologic spectrum, ranging from tumors with clearly visualized epithelial elements to heterologous tumors with non-epithelial elements like spindle cells, cartilage and bone [1-4]. With the help of immunohistochemical (IHC) markers, different morphologic entities within the larger group of metaplastic tumors have been identified. Among these is an unusual, "fibromatosis-like" metaplastic carcinoma. Currently, there is a limited understanding for this tumor as a result of its rarity [4,5]. We present a case of a "fibromatosis-like" metaplastic carcinoma associated with a micropapilloma in an elderly lady. This rare case is discussed to highlight its diagnostic and management issues.

## Case presentation

A 77-year-old lady presented with the complaints of a left-sided breast lump of 1-month duration. She had been a heart patient and had been on treatment for the last 4 years. On clinical examination a 3 × 2 cm firm, mobile, non-tender lump was identified in the outer quadrant of her left breast. The overlying skin of the breast along with nipple and areola were unremarkable. There was no significant axillary or cervical lymphadenopathy. The other breast was normal. She underwent a mammographic examination, followed by fine needle aspiration cytology (FNAC) that was essentially inconclusive. Subsequently, she underwent a frozen section for a primary diagnosis.

**Figure 1:**
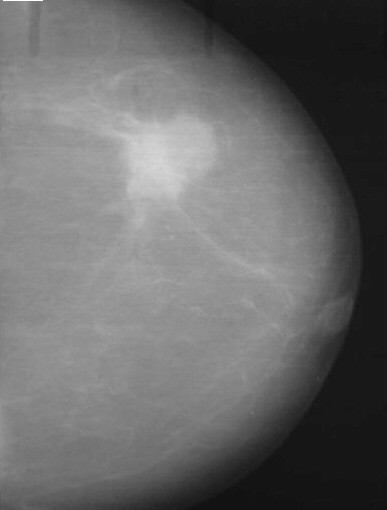
Mammographic findings. A bilobed soft tissue lesion measuring about 3.2 × 2.5 cms, suspicious for malignancy, seen in the upper and outer quadrant of the left breast.

On mammography, a 2 × 2 cm ill-defined mass with irregular margins was identified in the left upper outer quadrant. No micro-calcifications were seen. The right-sided breast was normal. (Figure 1).

### Pathological findings

The lumpectomy specimen on cut surface revealed a firm, grey-white, fibrous, un-encapsulated nodular tumor measuring 2 × 1.2 × 0.8 cm with infiltrative borders. No area of calcification was identified. The closest margin was the base and was found to be 0.5 cm away from the tumor.

### Microscopic findings

Frozen sections revealed a tumor with predominant spindle cells showing mild atypia, amidst a sclerotic stroma and conspicuously infiltrated the adjacent fat. A diagnosis of a low-grade sarcoma was favored over a metaplastic carcinoma. Therefore, a sentinel lymph node biopsy and/or an axillary node dissection (ALND) were not conducted at the time of surgery.

**Figure 2:**
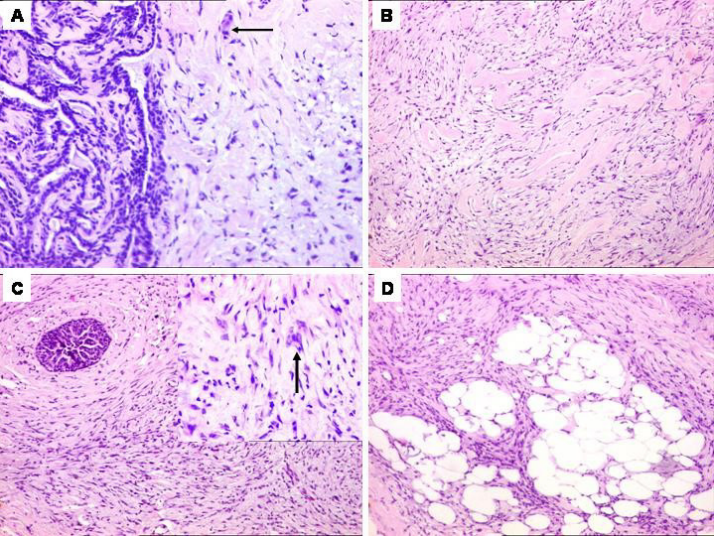
'Fibromatosis-like' carcinoma of the breast associated with a micropapilloma. A) Spindly tumor cells with an occasional cluster (arrow) in the vicinity of a micropapilloma. (H & E × 200). B). Interspersed areas of 'keloid-like' collagen, reminiscent of appearance of a fibromatosis. (H & E × 100). C). A focus of benign ductal hyperplasia amidst tumor cells. (H & E × 100). Inset showing spindle cells with focal cell clusters exhibiting minimal atypia. (H & E × 400). D). Tumor cells infiltrating the fat. (H & E × 100).

**Figure 3:**
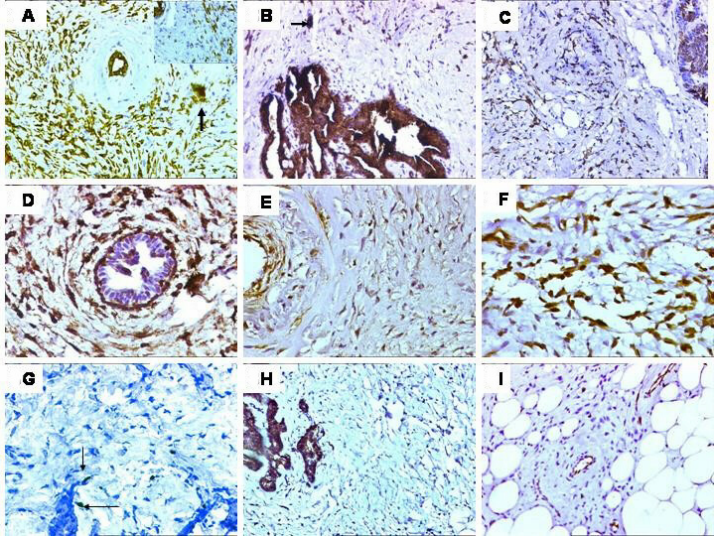
A). Tumor cells exhibiting strong cytokeratin (CK) expression (DAB × 200). **Inset **showing an occasional clusterof CK positive 'epithelial-like' cells. (DAB × 400). B). Tumor cellsadjacent to the micropapilloma showing positive CK7 expression. **Arrow **showing a cluster of cells (DAB × 100). C). Strongdiffuse expression for HMWCK. D). Positive vimentin expression. (DAB × 400). E). Focal expression for smooth muscle actin (SMA). A vessel identified in the proximity of tumor cells (DAB × 400). F). Positiveintranuclear p63 expression. (DAB × 400). G). Focal Ki-67 expression(arrows). (DAB × 400).. H). Negative expression for CD34 in the tumorcells. (DAB × 200).I. Negative CerbB-2/HER 2/neu expression. (DAB × 200).

Histological sections revealed a spindle cell tumor showing an infiltrative growth pattern with prominent areas of sclerosis reminiscent of keloid formation. The cells were mainly arranged in fascicles and displayed tapering nuclei with mild anisonucleosis. Mitoses were inconspicuous. Occasionally, the cells were plump with epithelioid shapes and revealed mild atypia with an occasional small cluster formation. Interspersed were foci of benign ductal hyperplasia and papillary hyperplasia, including a micropapilloma along with focal aggregates of chronic inflammatory cells. The micropapilloma did not show any significant atypia. (Figure 2A, 2B, 2C, 2D). No discrete squamous differentiation was identified. No focus of Ductal-carcinoma-*in-situ *(DCIS) was seen in any of the sections. The two closest differential diagnoses considered were fibromatosis and a "fibromatosis like" metaplastic carcinoma. A wide panel of IHC antibody markers was performed (Table 1). The tumor cells were simultaneously diffusely positive for epithelial markers i.e. the various cytokeratins CK, CK7, High molecular weight (HMWCK) and epithelial membrane antigen (EMA), along with a mesenchymal marker i.e. vimentin. (Figure 3A, 3B, 3C and 3D). All the cytokeratins were positive in the interspersed benign ducts that acted as internal controls. The tumor cells were negative for Gross cystic disease fluid protein (GCDFP), estrogen (ER) and progesterone receptor (PR). The myoepithelial markers i.e. smooth muscle actin (SMA) and p63 showed focal, positive expression. (Figure 3E, 3F). S100 and Desmin were negative. Ki-67 (proliferation marker) showed focal positivity in less than 5% tumor cells (Figure 3G). The tumor cells were negative for CD34 and CerbB-2/HER-2/neu. (Figure 3H, 3I). A diagnosis of a low-grade "fibromatosis-like" metaplastic carcinoma, associated with a micropapilloma, was finally made. All the cut margins were free of tumor.

**Table 1:** List and details of the IHC antibody markers.

**No**	**Antibody**	**Clone**	**Type**	**Dilution**	**Antigen retrieval**	**Source**
1	Cytokeratin (CK)	MNF116	Monoclonal	1:100	Enzymatic (Pronase)	Dako, Glostrup Denmark
2	CK7	OV-TL 12/30	Monoclonal	1:100	Microwave	Dako
3	High molecular weight (HMWCK)	34βE12	Monoclonal	1:50	Microwave	Dako
4	Epithelial membrane antigen (EMA)	E29	Monoclonal	1:100	Enzymatic (Pepsin)	Dako
5	Vimentin	V9	Monoclonal	1:50	Microwave	Dako
6	Gross cytic disease fluid protein (GCD-FP)	NCL-L-GCDFP-15	Monoclonal	1:50	Microwave	Novocastra
7	Estrogen receptor (ER)	ER1D5	Monoclonal	1:50	Pressure cooker	Dako
8	Progestrone receptor (PR)	PgR636	Monoclonal	1:50	Pressure cooker	Dako
9	Smooth muscle antigen (SMA)	1A4	Monoclonal	1:200	Enzymatic (Pepsin)	Dako
10	P63	GA4	Monoclonal	1:50	Microwave	Dako
11	S100	NA	Polyclonal	1:300	Enzymatic (Pepsin)	Dako
12	CD34	QBEnd10	Monoclonal	1:100	Microwave	Dako
13	Desmin	D33	Monoclonal	1:50	Microwave	Dako
14	Ki-67	MIB1	Monoclonal	1:50	Microwave	Dako
15	CerbB-2	CB11	Monoclonal	1:20	Microwave	Biogenex

NA: Not applicable

### Ultrastructural findings

A portion of fresh tumor tissue fixed in 3% glutaraldehyde was processed for electron microscopy. Ultra thin sections stained with uranyl acetate and lead citrate were observed under an electron microscope model: Zeiss 109, Germany.

**Figure 4:**
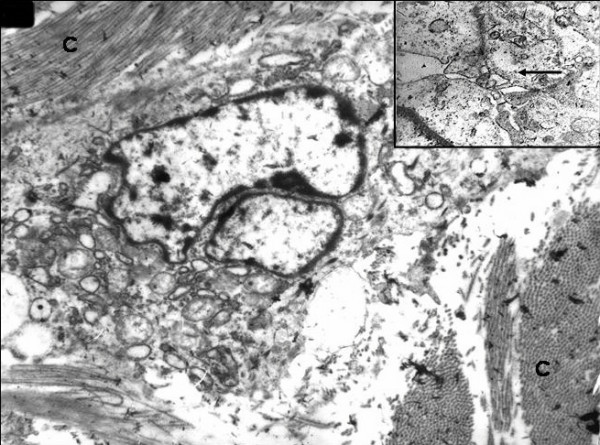
Ultrastructural appearance of the tumor cells. A single cell with peripheral villous processes embedded in a collagenous stroma. Magnification: 4,400×. **Inset **showing higher magnification of a tumor cell cluster with microvilli and cell junctions. Tumor cell exhibiting intracellular junctions (arrow). Magnification: × 20,000.

Ultrastructurally, the tumor cells embedded in a collagenous stroma showed fibroblastic and myoepithelial features along with presence of peripheral villous processes with a focal basal lamina and intercellular junctions (Figure 4).

### Follow-up

After surgery, the patient completed adjuvant radiotherapy (RT). Thereafter, she has been on a regular 2 monthly follow-up; including her metastatic work-up with Positron emission tomography (PET-CT) of the body and bone scan. Due to a high cardiac risk, a second surgery for an ALND was not performed. Nevertheless, till 1 year and 4 months of her follow-up she has not been identified with any lymphadenopathy, recurrent lesion or metastatic lesions in her body.

## Discussion

Spindle cell metaplastic breast tumors display a range of elements, including low-grade tumors to those with areas of high-grade sarcomas like fibrosarcomas and malignant fibrous histiocytomas [2-4]. Among these, a low-grade "fibromatosis-like" variant of a metaplastic carcinoma with only <5% tumor cells showing epithelial traits, has been a recently described entity [4,5]. Identification of this variant assumes importance as, like fibromatosis, it has more chances for local recurrence than distant metastasis [4]. At the same time its exact diagnosis from this close morphological differential i.e. fibromatosis and other benign and malignant spindle cell tumors has a far-reaching significance and requires application of IHC markers.

The present case observed in an elderly female who underwent a lumpectomy for a painless breast lump, exemplifies the diagnostic and management issues related to this tumor. The relatively bland nature of cells has prompted some authors to label it as a 'tumor' than a carcinoma [4].

This tumor has predilection for older women. In a classical premier series of 30 exclusive cases by Gobbi *et al *[4] and 24 cases by Sneige *et al *[6], the average age seen with this tumor was 63.4 years and 66 years respectively. Gobbi *et al *[4] identified these cases in women having a single palpable, generally painless, breast mass, more commonly in the left side (62.5%), as seen in our case.

On gross findings, they observed an average size of 2.7 cms with these tumors; mostly associated with unencapsulation and irregular borders [4]. In the present case, the tumor displayed an irregular and an infiltrative growth pattern, both grossly as well as microscopically; thereby closing mimicked an aggressive mesenchymal tumor/sarcoma on the frozen sections. In view of predominantly spindle cells with mild atypia, however, revealing prominent infiltration into the adjacent soft tissues, diagnosis of a low-grade sarcoma was favored over a metaplastic carcinoma that seemed to be the closest differential diagnosis. An exact diagnosis was not offered since multiple section examination and adjunctive IHC application were anticipated for arriving at a final diagnosis. However, in view of a favored diagnosis of a low-grade sarcoma, the patient was spared for an ALND. On histology, the differential diagnoses considered were nodular fasciitis (NF), an inflammatory myofibroblastic tumor, pseudoangiomatous stromal hyperplasia (PASH), a myofibroblastoma, a low-grade fibrosarcoma and of course fibromatosis, which was a very close differential diagnosis. Nodular fasciitis (NF) and an inflammatory myofibroblastic tumor were ruled out based on the lack of conspicuous lymphoplasmacytic infiltrates and myxoid background [7-9]. Absence of slit-like spaces lined by fibroblastic cells made a diagnosis of a pseudoangiomatous stromal hyperplasia (PASH) less likely. Lack of fascicular arrangements and lower cellularity helped to rule out a fibrosarcoma and pleomorphic sarcomas. [10]. The immunoprofile was also helpful in objectively ruling out the other mentioned differential diagnoses, all of which lack epithelial expression. Thus it is imperative to employ a panel of epithelial and myoepithelial markers in spindle cell tumors of breast. Unlike Schafernak *et al *[11], who noticed focal positivity for epithelial markers in the spindly cells, we observed a diffuse strong immunoreactivity in spindle and slightly more in plump cells as was observed by Gobbi *et al *[4]. Among the various cytokeratins, a diffuse strong expression of HMWCK that is believed to be an expression of squamous carcinomas was suggestive for metaplastic traits of the tumor. Similar finding have been noted by others [4,7]. As in our case, Gobbi *et al *[5] demonstrated CK7 positivity, mostly in glandular areas in both their cases of predominantly "fibromatosis-like" carcinomas that were analyzed with IHC. A positive co-expression of epithelial and mesenchymal markers in the spindly cells might be considered as suggestive of 'epithelial-mesenchymal' transition observed with these tumors.

Positive expression for myoepithelial markers in this case is further suggestive that these tumors might constitute as myoepithelial subtypes of a metaplastic carcinoma. A positive p63 expression, like in our case, has been lately identified in 86.3% cases of myoepithelial carcinomas in a study by Koker *et al *[12]. Ultrastructural findings further confirmed epithelial nature of these tumor cells with the presence of cell junctions and microvilli. Gersel *et al *[3] described a series of spindle cell carcinomas of the breast including deceptively bland carcinoma cells.

Micropapillomas and papillomas have been described to be associated with an increased risk for subsequent development of a breast carcinoma and may form its antecedent lesion [13]. Association of a micropapilloma with metaplastic carcinomas was also noted by Gobbi *et al *[5]. However, the 'fibromatosis-like' pattern was noted in only a few of their cases [5].

The optimal treatment options for this tumor remain unclear. However, most authors insist on a complete local treatment like lumpectomy with wide margins and adjuvant RT [4]. Rarely, such cases, generally larger sized, with distant metastasis have also been identified by Sneige *et al *[6] and Kinkor *et al *[14]. Though the present case limits an ALND in view of less likeliness for nodal metastasis, currently, given the epithelial nature of this tumor, it would be worthwhile to perform a sentinel lymph node dissection in such cases.

## Conclusion

An objective identification of this uncommon tumor with the help of a panel of IHC, along with the presence of a micropapilloma makes this case unique. Further, an uneventful follow-up for more than a year, despite avoidance of an axillary lymph node dissection suggests a relatively lesser metastatic potential of this tumor. A certain degree of index of suspicion in the pathologists' mind while dealing with spindle cell tumors of breast is helpful in making this diagnosis. Documentation of more of such cases with follow-up details would bring new light to the management and biological behavior of this unusual breast tumor.

## Conflict of interest

The author(s) declare that they have no competing interests.

## Authors' contributions

**BR**: Involved in the diagnosis of the case; design, preparation and drafting of the manuscript.

**TMS**: Involved in the diagnosis; preparation of the manuscript and in the ultrastructural analysis.

**RDB**: Treating breast oncosurgeon, provided the clinical and follow-up details.

**RFC**: Overall supervision and has given the final approval of the manuscript.
